# P-Cadherin Expression in Feline Mammary Tissues

**DOI:** 10.1155/2012/687424

**Published:** 2012-10-03

**Authors:** Ana Catarina Figueira, Ana Sofia Teodósio, Júlio Carvalheira, Manuela Lacerda, Augusto de Matos, Fátima Gärtner

**Affiliations:** ^1^Escola Universitária Vasco da Gama (EUVG), Quinta de S. Jorge, Estrada da Conraria, Castelo Viegas, 3040-714 Coimbra, Portugal; ^2^Instituto de Ciências Biomédicas Abel Salazar, Universidade do Porto (ICBAS-UP), Rua de Jorge Viterbo Ferreira No. 228, 4050-313 Porto, Portugal; ^3^Instituto Português de Oncologia de Coimbra Francisco Gentil, EPE (IPOCFG,EPE) Avenida Bissaya Barreto, No. 98, 3000-075 Coimbra, Portugal; ^4^Instituto de Patologia e Imunologia Molecular da Universidade do Porto (IPATIMUP), Rua Dr Roberto Frias s/n, 4200-465 Porto, Portugal

## Abstract

The search for molecular markers in the feline mammary gland, namely, the adhesion molecules belonging to the cadherin family, is useful in the understanding of the development of mammary carcinomas in felines and humans. To study P-cadherin expression in the feline mammary gland, 61 samples of normal (*n* = 4), hyperplastic (*n* = 12), and neoplastic (*n* = 45) feline mammary tissues were examined. In both normal and hyperplastic mammary tissues as well as in benign tumours, P-cadherin immunolabelling was restricted to myoepithelial cells. In malignant tumours, however, there was an aberrant epithelial P-cadherin immunoexpression in 64.1% (*n* = 25) of cases, with a membranous and/or cytoplasmic pattern of distribution. A statistically significant relationship was seen between epithelial P-cadherin expression and malignant mammary lesions (*P* = 0.0001). In malignant mammary tumours, there was likewise a statistically significant relationship between aberrant P-cadherin immunoexpression and histological grade (*P* = 0.0132). Aberrant epithelial P-cadherin expression seems to be related to malignancy in the feline mammary gland. To confirm the results of this investigation, further studies with larger samples and follow-up studies are warranted.

## 1. Introduction 

Cadherins are elementary membrane glycoproteins that mediate calcium-dependent cellular adhesion [1–4] and play an important role in the formation and maintenance of normal tissue architecture [2–4]. Placental cadherin (P-cadherin) is a classical cadherin [5] expressed by myoepithelial cells [6–11] of the human and canine mammary gland. Changes in P-cadherin expression have been observed and implicated in human breast carcinogenesis [10, 12–17].

Mammary tumours are the third most common neoplasm in felines. They show similarities with human breast tumours, including histological characteristics and clinical evolution, and thus have been proposed as an excellent animal model for the study of mammary carcinogenesis [18].

The present work examines the P-cadherin immunoreactivity in feline mammary tissues and its relationship with the histological grade and type of feline mammary tumours.

## 2. Material and Methods

### 2.1. Specimens

Feline mammary hyperplasias (*n* = 12), benign mammary tumours (*n* = 6), malignant mammary tumours (*n* = 39), and lymph node metastases (*n* = 3) samples were obtained from the archives of the veterinary pathology laboratory of the “*Instituto de Ciências Biomédicas Abel Salazar*” of Porto University, Portugal, and normal mammary gland samples (*n* = 4) were collected at necropsy in the same laboratory. The mammary gland lesions were originally collected from surgical excision of feline mammary masses. Tissues were fixed in 10% buffered formalin, dehydrated and embedded in paraffin wax. Sections 3 *μ*m thick were cut from each block and stained with both haematoxylin and eosin (HE) for routine histological examination, or processed and stained for immunohistochemistry.

### 2.2. Histological Examination 

Lesions were classified by two independent pathologists (FG and ML) from HE stained sections, using the World Health Organization criteria for the classification of tumours in domestic animals [19] (Table 1). If a sample contained more than one type of pattern, the classification was made according to the predominant pattern, as suggested by Misdorp et al., [19]. 

Malignant epithelial tumours were graded according to the Elston-Ellis modification of the Bloom and Richardson grading system (Nottingham grading system) for human breast carcinomas [20]. The histological grade was based on the assessment of three morphological features: tubule formation, nuclear pleomorphism, and mitotic counts. Each feature was assessed and scored on a scale of 1–3 (slight, moderate, or marked) for a possible total of 3–9 points. For each tumour, the total score indicates the histological grade (1–3). The histological grade was allocated by an arbitrary division of the total points: grade 1 (well differentiated) 3, 4 or 5 points; grade 2 (moderately differentiated) 6 or 7 points; and grade 3 (poorly differentiated) 8 or 9 points (Table 2).

### 2.3. Immunohistochemistry

P-cadherin immunoexpression was evaluated on sequential 3 *μ*m sections of each sample on Dako REAL Capillary Gap Microscope Slides, Code. S2024 (75 *μ*m). The sections were dried in an oven at 56°C overnight. The tissues were deparaffinized, rehydrated, and treated with EDTA buffer (Zymed Ref. no. 00-5500, EDTA solution pH 8, 20x concentrate) for 2 cycles of 10 minutes at 900 W in a microwave oven for antigen retrieval. The immunohistochemical assay was performed using the modified avidin-biotin-peroxidase complex (ABC) method with a commercial detection system (Dako REAL Detection System Streptavidin Peroxidase (HRP) Dako REAL Detection System, Peroxidase/DAB+, Rabbit/Mouse, Code K5001). The slides were developed in an automated slide staining system (DAKO TechMateTM 500 Plus Biotek Solutions) in the histopathology laboratory of the “*Instituto Português de Oncologia de Coimbra Francisco Gentil*”, Coimbra, Portugal. A specific mouse antihuman monoclonal antibody against P-cadherin was used. The antibody (diluted 1 : 50) was directed at the extracellular domain of this adhesion molecule (clone 56, BD Transduction Laboratories, Lexington, Ky, USA).

Complementary monoclonal antibodies to assess the epithelial or myoepithelial phenotype of the cells were used on successive serial sections of selected samples. A specific mouse antihuman monoclonal antibody against cytokeratin AE1/AE3 (AE1/AE3, Chemicon International, Temecula, USA), diluted 1 : 1500, was used as an epithelial marker. A specific mouse antihuman monoclonal antibody against p63 protein (clone 4A4, Lab Vision Corporation - NeoMarkers, Fremont, USA), diluted 1 : 150, was used as a myoepithelial marker. Antigen retrieval for these antibodies was carried out by microwave oven treatment for 2 cycles of 10 minutes at 900 W in a 10 nM citrate buffer, pH 6.0. 

Human nipple was used as positive control for P-cadherin and p63 protein antibodies. Human appendix was used as positive control for AE1/AE3 antibody. For negative controls, the primary antibody was replaced by mouse IgG1. Adjacent normal mammary tissues or skin were used as internal positive controls.

### 2.4. Evaluation of P-Cadherin Immunolabelling

The assessment of P-cadherin expression in feline mammary tissues was based on a semiquantitative analysis [8], according to the percentage of immunoreactive luminal epithelial cells, with a membranous and/or cytoplasmatic pattern of cellular distribution (aberrant pattern). The following scores were established to classify the tissue aberrant immunoexpression: 0: <10% positive cells (considered negative for P-cadherin immunostaining); 1: 10–25% positive cells; 2: 26–50% positive cells; 3: >50% positive cells.

### 2.5. Statistical Methods

Contingency tables and likelihood ratio chi-square test were used, with SAS/STAT, 1989 (SAS Institute Inc., Cary, NC, USA) and, in all instances, significance was set at *P* < 0.05.

## 3. Results

### 3.1. P-Cadherin Expression

The immunohistochemical results for P-cadherin expression are summarized in Table 3. 

**Figure 1:**
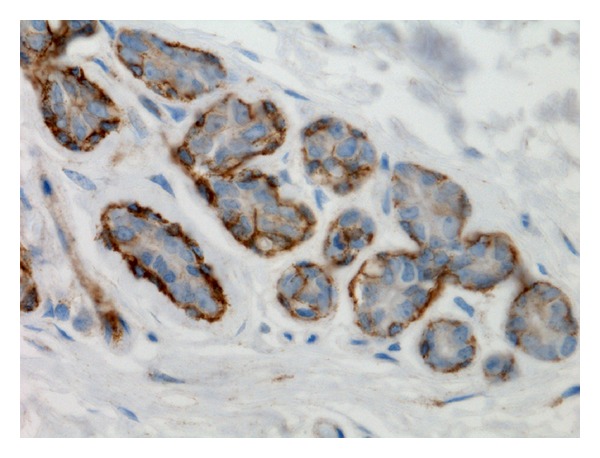
Normal mammary gland. Membranous and cytoplasmic P-cadherin immunoreactivity in myoepithelial cells (streptavidin-biotin-peroxidase method, 630x).

**Figure 2:**
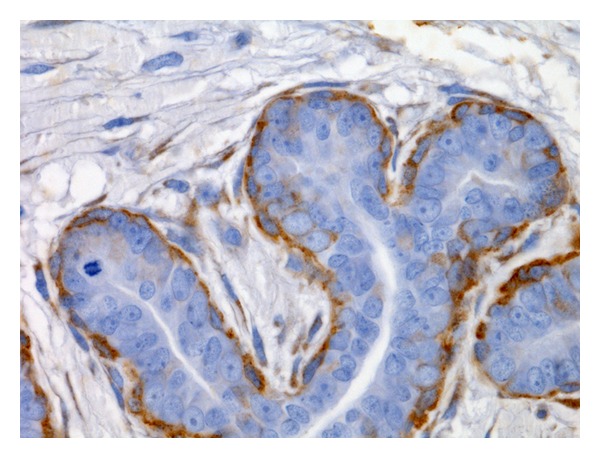
Fibroadenomatous change. Membranous and cytoplasmic P-cadherin immunoreactivity in myoepithelial cells (streptavidin-biotin-peroxidase method, 630x).

In normal nonlactating mammary glands, P-cadherin immunolabelling was restricted to myoepithelial cells (Figure 1). Hyperplastic mammary tissues showed a P-cadherin immunoexpression limited to myoepithelial cells (Figure 2), similar to the pattern observed in normal nonlactating mammary glands. 

**Figure 3:**
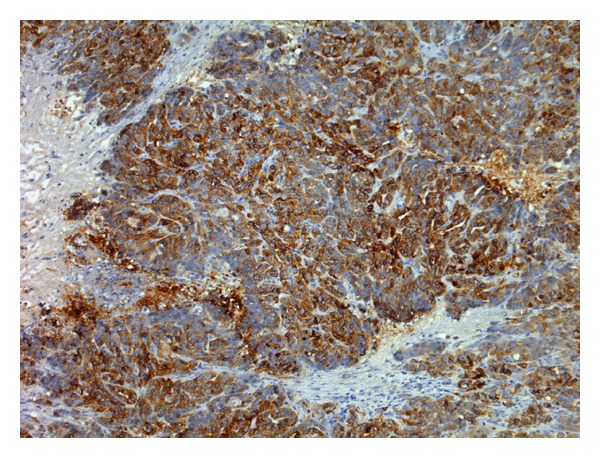
Solid carcinoma. Aberrant P-cadherin immunoreactivity in luminal epithelial cells (membranous and cytoplasmic) (streptavidin-biotin-peroxidase method, 100x).

**Figure 4:**
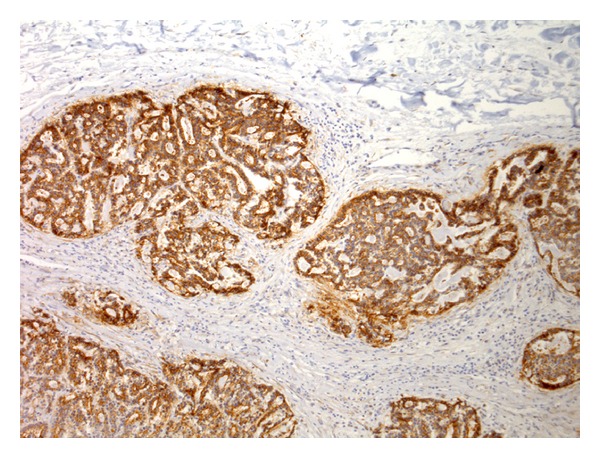
Tubulopapillary carcinoma. Aberrant P-cadherin immunoreactivity in luminal epithelial cells (membranous and cytoplasmic) (streptavidin-biotin-peroxidase method, 100x).

**Figure 5:**
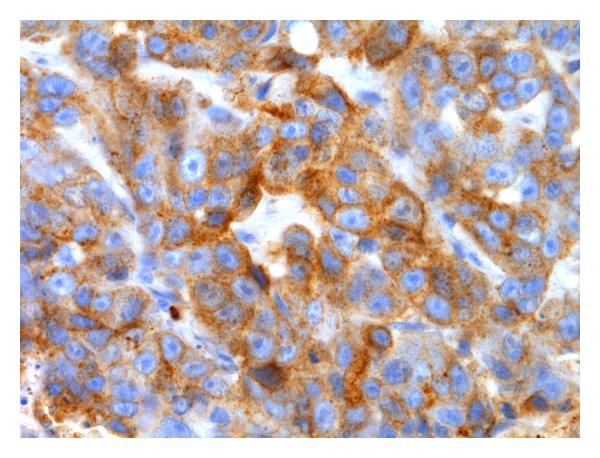
Solid carcinoma. Membranous and granular cytoplasmic aberrant P-cadherin immunoreactivity in luminal epithelial cells (streptavidin-biotin-peroxidase method, 630x).

**Figure 6:**
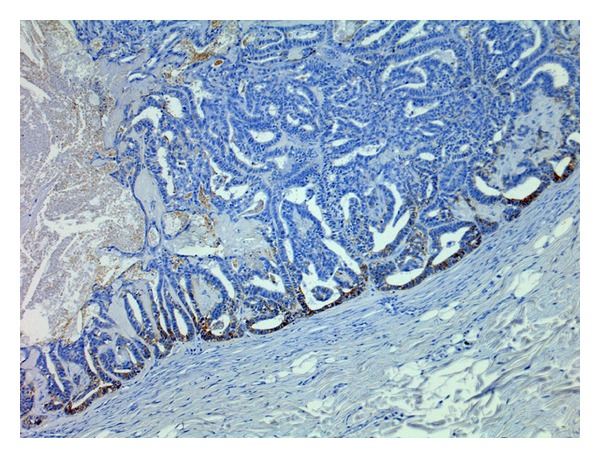
Tubulopapillary carcinoma. Strong aberrant P-cadherin immunoreactivity in the periphery of the tumour (streptavidin-biotin-peroxidase method, 100x).

In benign tumours, P-cadherin immunoexpression was restricted to myoepithelial cells while in malignant tumours it was observed in luminal epithelial cells (membranous and/or cytoplasmic). This aberrant expression was observed in two thirds of malignant mammary tumours, and two thirds (*n* = 15) of the positive ones had immunolabelling in more than one fourth of the cells (Figures 3 and 4). The cytoplasmic immunolabelling often showed a granular pattern (Figure 5). In eight malignant mammary tumours, P-cadherin immunostaining differed from area to area with a greater intensity and higher number of positive cells on the periphery of the tumour (Figure 6). Both solid and tubulopapillary malignant tumours showed aberrant expression of P-cadherin, while the single cribriform carcinoma showed negative immunostaining for P-cadherin.

Three lymph node metastases were studied (two from grade 1 tubulopapillary carcinomas and one from a grade 3 solid carcinoma). One metastasis from a solid carcinoma showed 10–25% of cells expressing P-cadherin, while in the primary tumour more than 50% of the cells were immunoreactive. Both primary tubulopapillary carcinomas had 10–25% P-cadherin positive cells, while more than 50% of tumour cells in one lymph node and 25–50% in the other node expressed P-cadherin. Thus lymph node metastases from the tubulopapillary carcinomas had higher aberrant P-cadherin immunoexpression than the primary tumours, while the opposite was seen in the solid carcinoma.

In normal and hyperplastic mammary glands as in benign mammary tumours there was positive immunolabelling for AE1/AE3 and p63, showing two different cell phenotypes, epithelial, and myoepithelial, respectively. In malignant mammary tumours there was only one type of cells, the luminal epithelial cells evidenced by AE1/AE3 immunoreactivity and there was no immunoreactivity to p63.

### 3.2. Relationship between Aberrant P-Cadherin Expression, Histological Classification, and Tumour Grade

Benign and malignant tumours diverged significantly with respect to aberrant P-cadherin immunoexpression (*P* < 0.0001) (Table 4). No significant statistical relationship between P-cadherin immunoexpression and malignant histological type was observed, but a significant statistical relationship was observed between P-cadherin immunoexpression and the histological grade of malignant tumours (*P* = 0.0132) (Table 5). All grade 3 malignant tumours (poorly differentiated) showed more than 25% P-cadherin immunopositivity while most grades 1 and 2 showed less than 25% P-cadherin immunopositivity.

## 4. Discussion

Cadherins are cell adhesion molecules important in the morphogenesis and maintenance of the normal tissue architecture. The control of cellular adhesion and motility is one of the crucial mechanisms responsible for the initiation and progression of tumours [21]. P-cadherin is a classical cadherin that has been implicated in human mammary carcinogenesis. Several studies on its immunoexpression have demonstrated an association with biological aggressiveness [10, 12, 14–17, 22], since P-cadherin is frequently overexpressed in high-grade tumors being considered as a marker of poor prognosis in human breast cancer [12, 17, 23]. P-cadherin has been described as an inducer of cancer cell migration and invasion; the molecular mechanisms underlying this process seem to be related with signaling mediated by the P-cadherin juxtamembrane domain, the P-cadherin soluble form and the secretion of matrix metalloproteases [23–25]. 

Considering feline mammary tumours as promising models to achieve a better understanding of human breast carcinogenesis, P-cadherin was studied as a prognostic marker in these neoplasms. Immunohistochemical techniques were performed using an automated system which, according to Cassali et al. [26], results in improved quality, reproducibility, speed, and standardization. 

In the present study, normal and hyperplastic mammary tissues, as well as benign lesions, showed P-cadherin immunoexpression similar to that described in human mammary tissues [10, 12, 27], with immunoreactivity in the basal cell layer of ductal and alveolar structures, that correspond morphologically to the myoepithelium. This is similar to the pattern described by other authors in normal and hyperplastic canine mammary tissues [8, 28]. Feline benign tumours were negative for epithelial P-cadherin expression (aberrant pattern), in opposition to canine benign mammary tumours, where Gama et al. [8] observed an aberrant epithelial expression in 43%, with 10 to 25% immunoreactive cells. 

P-cadherin is not expressed in epithelial cells of the normal mammary gland. However in feline malignant mammary tumours, this molecule was aberrantly detected in epithelial cells. Sixty four percent (*n* = 25) of the feline malignant mammary tumours showed aberrant P-cadherin expression in luminal epithelial cells. This expression pattern is in accordance with previous studies of human breast cancer [10, 12–14, 16, 17, 29] and canine mammary tumours [8]. In fact, Gama et al. [8] observed aberrant P-cadherin epithelial immunoexpression in 64% of the studied canine malignant tumours, a percentage similar to that observed in the present study. As observed in women [14, 17] P-cadherin has an membranous immunostaining pattern, generally associated with cytoplasmic staining as well. 

Peralta Soler et al., [29] demonstrated that P-cadherin immunoexpression in human breast carcinomas was mostly in cells at the periphery of the tumour and in invading nests. Likewise, in our study we observed a higher P-cadherin expression in the outside edge of the tumours. 

P-cadherin has been considered a progression marker of human breast cancer [12]. Some theories have been proposed to explain its aberrant expression [12, 29–31] and the mechanisms underlying the invasive behavior of P-cadherin-expressing breast tumours [25]. The biological meaning of this immunoexpression in mammary carcinogenesis, however, is still poorly understood. 

In feline normal mammary gland, P-cadherin is expressed only by the myoepithelial cells, suggesting that the presence of this molecule in feline mammary malignant tumours could point to a myoepithelial differentiation. We used the molecular marker p63 protein to explore this hypothesis since p63 is described as a highly sensitive and specific myoepithelial cell marker in canine [32, 33] and human breast tissues [34, 35], while the pancytokeratin AE1/AE3 was applied as an epithelial cell marker [8, 33, 36]. 

In the normal feline mammary glands, as well as in hyperplastic lesions and benign tumours, p63 and P-cadherin were coexpressed in the same cells, showing that P-cadherin was expressed exclusively by myoepithelial cells in these tissues. In malignant mammary tumours, however, P-cadherin positive cells were negative for p63 and positive for AE1/AE3. These results are in accordance with those described in canine [8] and human [10, 12] mammary tumours and demonstrate that, in feline mammary carcinomas, the expression of P-cadherin cannot be considered a marker of myoepithelial differentiation. 

No relationship was seen between the tumours histological type and P-cadherin expression, contrary to the findings in canine [8] and human mammary tumours [10, 11]. 

In this study, P-cadherin immunoexpression in neoplastic epithelial cells was observed exclusively in malignant mammary tumours and this aberrant expression was strongly related with histological grade (*P* = 0.0001), as described in canine mammary tumours [8, 9]. In fact, all grade 3 carcinomas expressed P-cadherin by more than one half of its cells. Studies in human breast cancer demonstrated a significant correlation between P-cadherin expression and high histological grade [10, 12–17, 29], and one study [13] revealed that most of the P-cadherin expressing carcinomas were grade 3.

In women with breast cancer, the aberrant expression of P-cadherin has been associated with aggressive behaviour [14, 15] and worse prognosis [4, 11, 14, 15, 17, 22, 23, 29, 37–39], and thus may represent a promising antibody therapeutic target [22].

This study in feline mammary carcinomas suggests a relationship between aberrant immunoexpression of P-cadherin, malignant phenotype, and histological grade. However, these results should be confirmed with a greater number of cases and to conclusively define aberrant P-cadherin immunoexpression as a marker of biological aggressiveness and a prognostic indicator, it will be necessary to study larger populations in prospective follow-up studies.

## Figures and Tables

**Table 1:** Histological classification of mammary lesions and their absolute and relative frequencies.

Histological classification	*n*	%
Hyperplasias (*n* = 12)		
Lobular hyperplasia	3	5.26
Fibroadenomatous change	9	15.80
Benign tumours (*n* = 6)		
Simple adenoma	2	3.51
Fibroadenoma	3	5.26
Duct papilloma	1	1.75
Malignant tumours (*n* = 39)		
Tubulopapillary carcinoma	18	31.58
Solid carcinoma	20	35.09
Cribriform carcinoma	1	1.75

**Table 2:** Histological grade of the malignant epithelial tumours.

Histological type	*n*	Grade 1	Grade 2	Grade 3
		*n* (%)	*n* (%)	*n* (%)
Tubulopapillary carcinoma	18	14 (77.78%)	3 (16.67%)	1 (5.55%)
Solid carcinoma	20	3 (15.00%)	13 (65.00%)	4 (20.00%)
Cribriform carcinoma	1	0 (0.00%)	1 (100.00%)	0 (0.00%)

Total	39	17 (43.59%)	17 (43.59%)	5 (12.82%)

**Table 3:** Aberrant P-cadherin immunoreactivity in feline mammary tissues.

Mammary tissues	*n*	Aberrant P-cadherin immunoreactivity			
		0	1	2	3
Normal mammary gland	4	4 (100.00%)	0 (0.00%)	0 (0.00%)	0 (0.00%)
Lobular hyperplasia	3	3 (100.00%)	0 (0.00%)	0 (0.00%)	0 (0.00%)
Fibroadenomatous change	9	9 (100.00%)	0 (0.00%)	0 (0.00%)	0 (0.00%)
Simple adenoma	2	2 (100.00%)	0 (0.00%)	0 (0.00%)	0 (0.00%)
Fibroadenoma	3	3 (100.00%)	0 (0.00%)	0 (0.00%)	0 (0.00%)
Duct papilloma	1	1 (100.00%)	0 (0.00%)	0 (0.00%)	0 (0.00%)
Tubulopapillary carcinoma	18	6 (33.33%)	5 (27.78%)	4 (22.22%)	3 (16.67%)
Solid carcinoma	20	7 (35.00%)	5 (25.00%)	4 (20.00%)	4 (20.00%)
Cribriform carcinoma	1	1 (100.00%)	0 (0.00%)	0 (0.00%)	0 (0.00%)

Total	61	36 (59.02%)	10 (16.39%)	8 (13.11%)	7 (11.48%)

0: <10%; 1: 10–25%; 2: 25–50%; 3: >50%.

**Table 4:** Relationship between aberrant P-cadherin immunoexpression and mammary lesions.

Mammary lesions	*n*	Aberrant P-cadherin immunoreactivity				*P*
		0	1	2	3
Hyperplasias	12	12 (100.00%)	0 (0.00%)	0 (0.00%)	0 (0.00%)	*P* = 0.0001
Benign tumours	6	6 (100.00%)	0 (0.00%)	0 (0.00%)	0 (0.00%)	
Malignant tumours	39	14 (35.90%)	10 (25.64%)	8 (20.51%)	7 (17.95%)	

Total	57	32 (56.14%)	10 (17.54%)	8 (14.04%)	7 (12.28%)	

0: <10%; 1: 10–25%; 2: 25–50%; 3: >50%.

**Table 5:** Relationship between aberrant P-cadherin immunoexpression and histological grade of malignant tumours.

Histological grades	*n*	Aberrant P-cadherin immunoreactivity				*P*
		0	1	2	3
Grade 1	17	4 (23.53%)	6 (35.29%)	4 (23.53%)	3 (17.65%)	*P* = 0.0132
Grade 2	17	10 (58.82%)	4 (23.53%)	2 (11.76%)	1 (5.88%)	
Grade 3	5	0 (0.00%)	0 (0.00%)	2 (40.00%)	3 (60.00%)	

Total	39	14 (35.90%)	10 (25.64%)	8 (20.51%)	7 (17.95%)	

0: <10%; 1: 10–25%; 2: 25–50%; 3: >50%.
